# Evolving Bioprosthetic Tissue Calcification Can Be Quantified Using Serial Multislice CT Scanning

**DOI:** 10.1155/2013/617329

**Published:** 2013-09-08

**Authors:** B. Meuris, H. De Praetere, W. Coudyzer, W. Flameng

**Affiliations:** ^1^Department of Cardiovascular Diseases, Katholieke Universiteit Leuven, 3000 Leuven, Belgium; ^2^Department of Radiology, Katholieke Universiteit Leuven, 3000 Leuven, Belgium

## Abstract

*Background.* We investigated the value of serial multislice CT scanning for *in vivo* determination of evolving tissue calcification in three separate experimental settings. *Materials and Methods.* Bioprosthetic valve tissue was implanted in three different conditions: (1) glutaraldehyde-fixed porcine stentless conduits in pulmonary position (*n* = 6); (2) glutaraldehyde-fixed stented pericardial valves in mitral position (*n* = 3); and (3) glutaraldehyde-fixed pericardial tissue as patch in the jugular vein and carotid artery (*n* = 16). Multislice CT scanning was performed at various time intervals. *Results.* In stentless conduits, the distribution of wall calcification can be reliably quantified with CT. After 20 weeks, the CT-determined mean calcium volume was 1831 ± 581 mm³, with a mean wall calcium content of 89.8 ± 44.4 **μ**g/mg (*r* ^2^ = 0.68). In stented pericardial valves implanted in mitral position, reliable determination of tissue mineralization is disturbed by scattering caused by the (continuously moving) alloy of the stent material. Pericardial patches in the neck vessels revealed progressive mineralization, with a significant increase in mean HU and calcium volume at 8 weeks after implantation, rising up to a level of 131.1 ± 39.6 mm³ (mean calcium volume score) and a mean calcium content of 19.1 ± 12.3 **μ**g/mg. *Conclusion.* The process of bioprosthetic tissue mineralization can be visualized and quantified *in vivo* using multislice CT scanning. This allows determination of the kinetics of tissue mineralization with intermediate *in vivo* evaluations.

## 1. Introduction

Calcific degeneration of implanted bioprosthetic material is still problematic. Despite several advances in tissue treatment and valve design, tissue calcification remains the most important factor limiting the durability of biological heart valves [1]. New valve designs or new tissue treatments are tested in chronic animal models to evaluate whether the durability can be enhanced and the calcification potential can be diminished. Several animal models, ranging from simple subcutaneous implants in rats to whole valve implantations in sheep, exist to evaluate the behavior of chronically implanted tissue [2]. In all currently used models, evaluation of the extent of tissue mineralization is performed at the end of the experiment. Depending on the model, a bioprosthetic tissue fragment or a complete valve is implanted during a certain predetermined time, after which the animal is sacrificed, and the explanted tissue is recovered for further analysis. The explanted tissue can be investigated using different techniques such as X-ray analysis, histology, immunohistochemistry, and calcium content determination. This experimental strategy has proven to deliver reliable and reproducible results [3, 4]. However, it delivers only information about the end stage of tissue calcification; there is no information on tissue mineralization during the implant period. In simplified animal models, efforts have been made to overcome this lack of “intermediate” information by performing “staged” explantations in time, where implanted tissue is recovered after 1 week, 4 weeks, 8 weeks, and so forth [5]. There are two drawbacks to this strategy. This approach is only feasible in low-cost, small animal models where tissue patches are implanted (usually subcutaneous in rats), since performing valve implantations in large animals is too complicated and expensive to engage in high-volume experiments with “staged” explantations in time. Unfortunately, the reliability of small animal models is inferior to large animal experiments [6]. Secondly, one still is confronted with an interindividual variability between animals. There is no direct comparison between different stages of tissue calcification within one animal. In the current study, we investigated the potential use of multislice CT scanning for *in vivo* visualization and quantification of biological tissue mineralization. Three separate experimental models were explored in juvenile sheep: (1) stentless porcine valve implantation as right ventricular outflow tract (RVOT) substitute; (2) stented pericardial valve implantation in mitral position, and (3) implantation of pericardial tissue patches into the jugular vein and carotid artery. All animals were subjected to serial multislice CT scanning, repeated at fixed time intervals. Goal of the study was to detect and quantify tissue mineralization *in vivo*, using noninvasive methodology.

## 2. Materials and Methods

### 2.1. Animal Models

Eleven Lovenaar sheep between 8 and 11 months of age (weighing 43 ± 5 kg) were used in the study. The animals were bred at a special unit of the KU Leuven and procured for research by the Animalium KU Leuven. The experiments were approved by the Ethics Committee of the Katholieke Universiteit Leuven. All animals were female. The animals were premedicated with ketamine (10–20 mg/kg intramuscularly), and anesthesia was induced with increasing concentrations of isoflurane in oxygen. Anesthesia was maintained with isoflurane in 5 L/min O_2_ and 2 L/min N_2_O. Three different experimental settings were used.

- *Implantation of Stentless Xenografts in Pulmonary Position (RVOT Model, n* = 6*).* The implantation procedure in the RVOT model was described before [7]. A left thoracotomy and exposure of the left carotid artery and jugular vein were performed. After administration of heparin (3 mg/kg), the animal was placed on cardiopulmonary bypass (CPB) using the neck vessels. The main pulmonary artery was cross-clamped, the native pulmonary valve excised, and the pulmonary root replaced by a stentless xenograft (Edwards PrimaPlus valves, Edwards Lifesciences, Irvine, California). After weaning from bypass, the chest and surgical wounds were closed, the mechanical ventilation was discontinued, and the animal was allowed to recover. Implant duration was set at 20 weeks, with CT scanning performed at two time intervals: after 12 weeks and 20 weeks (prior to explantation).
- *Implantation of Stented Xenografts in Mitral Position (Mitral Model, n* = 3*).* The implantation procedure in the mitral model was described before [2]. Using a similar methodology for thoracotomy and CPB application, the left atrium was opened under induced ventricular fibrillation. A stented, porcine pericardial valve (experimental glutaraldehyde-fixed valve, no additional treatment, provided by Edwards Lifesciences) was implanted using separate sutures. The anterior leaflet of the native mitral valves was excised; the remainder stayed in situ. Implant duration was set at 20 weeks, with CT scanning performed at four time intervals: after 1, 2, 3, and 4 months (with the last scan between 16 and 20 weeks, prior to explantation of the valve).
- *Implantation of Pericardial Patches into the Neck Vessels (Patch Model, n* = 2*, 16 Separate Patches).* The implantation procedure in the patch model was described before [6, 8]. Two glutaraldehyde-fixed pericardial patches, each sized 13 × 13 mm, were implanted into the jugular vein on both sides (meaning 4 patches in venous position in each animal) and two patches sized 7 × 7 mm were implanted into the carotid artery on both sides (meaning 4 patches in arterial position in each animal). Implant duration was set at 20 weeks, with CT scanning performed at four time intervals: after 1, 2, 3, and 4 months (with the last scan between 16 and 20 weeks, prior to explantation of the patches). To allow identification of the implanted samples on the CT scans, small titanium markers (99% pure titanium, size 1 × 1 mm) were implanted in between all patches.

### 2.2. Multislice CT Scanning

Serial CT scanning was performed using a Siemens Somatom Sensation 64-slice CT scan. The animals were sedated using an intramuscular injection of ketamine, followed by mask ventilation with isoflurane (no intubation and no mechanical ventilation). The obtained CT images were analyzed further with 3D reconstructions. For detection and automated quantification of calcification, cutoff values were set at 100 and 400 Hounsfield units (HU), meaning that all densities >100 HU and <400 HU were considered to be pathologically mineralized tissue. Within the predetermined area of the implanted sample, the mean HU was measured as well as the total volume of calcium (in mm³, as determined by the cutoff values of >100 and <400 HU). In the RVOT model and mitral model, the predetermined area was the area of the valve. In the patch study, the predetermined area was defined based on the location of the implanted titanium markers.

### 2.3. Explantation and Analysis

After 20 weeks, the animals were again anesthetized and sacrificed (after full heparinization) by an overdose pentobarbital and KCL intravenously. The implanted bioprosthesis or patches were excised and analyzed as follows.

- Explant gross examination. After careful rinsing, gross examination of the explanted specimen was performed with special attention to vegetations, hematoma, thrombosis, stiffness of the wall portion, visible calcifications, retraction, tissue overgrowth (pannus), and leaflet tears or perforations.
- X-ray examinations were performed (Faxitron). The stentless valves were examined first in a horizontal plane perpendicular to the inflow-outflow axis showing the leaflets. The root was then longitudinally sectioned through the commissures providing three wall portions each containing one leaflet. The 3 sections were unrolled in a single plane and X-rays were taken. Also the stented valves and explanted patches were X-rayed.
- Half of every tissue specimen was used for calcium determination and the remains for histology. For histology, five *μ*m thick sections were prepared. The sections were embedded in paraffin and stained with hematoxylin and eosin and Von Kossa staining for calcium.
- For quantification of the calcium content the samples were lyophilized, weighed, and dissolved in 20% hydrochloric acid solution (10 mg dried tissue/mL HCl) for 24 hours. After homogenization, samples were kept at 70°C overnight. Samples were then analyzed with a Calcium kit (Chema Diagnostica, Monsano, Italy) and a spectrophotometer (Multiscan EX, Thermo Electron Corp, Woburn, Mass). All values are expressed in *μ*g/mg dry weight (DW). For the RVOT model, the obtained calcium content values (in *μ*g/mg) were used to estimate the “absolute” calcium content of the whole valve by correcting for the weight of the explanted valve (in mg). This calculation was made to determine the correlation with the calcium content values as measured by the CT scan (that take the whole valve into account).

### 2.4. Statistical Analysis

Data are expressed as mean ± standard deviation (SD). Standard *t*-test was used for comparison between groups. Linear regression analysis was used for comparison between CT-based estimated calcium contents and actual measured calcium content values after explantation. For the analysis of the sequential CT-based values over time, a linear mixed model was used with time points as repeated measurements. Statistica version 9 was used.

## 3. Results

All animals survived the operative procedure well. One animal in the mitral model developed endocarditis and died early. The implanted valve could not be used for further analysis. All other animals stayed alive during the planned implantation period. The scheduled CT scans (at 12 and 20 weeks in the RVOT model and at 1, 2, 3, and 4 months in both other models) were carried out without problems.

### 3.1. RVOT Model (Stentless Valves in RVOT Position)

**Figure 1:**
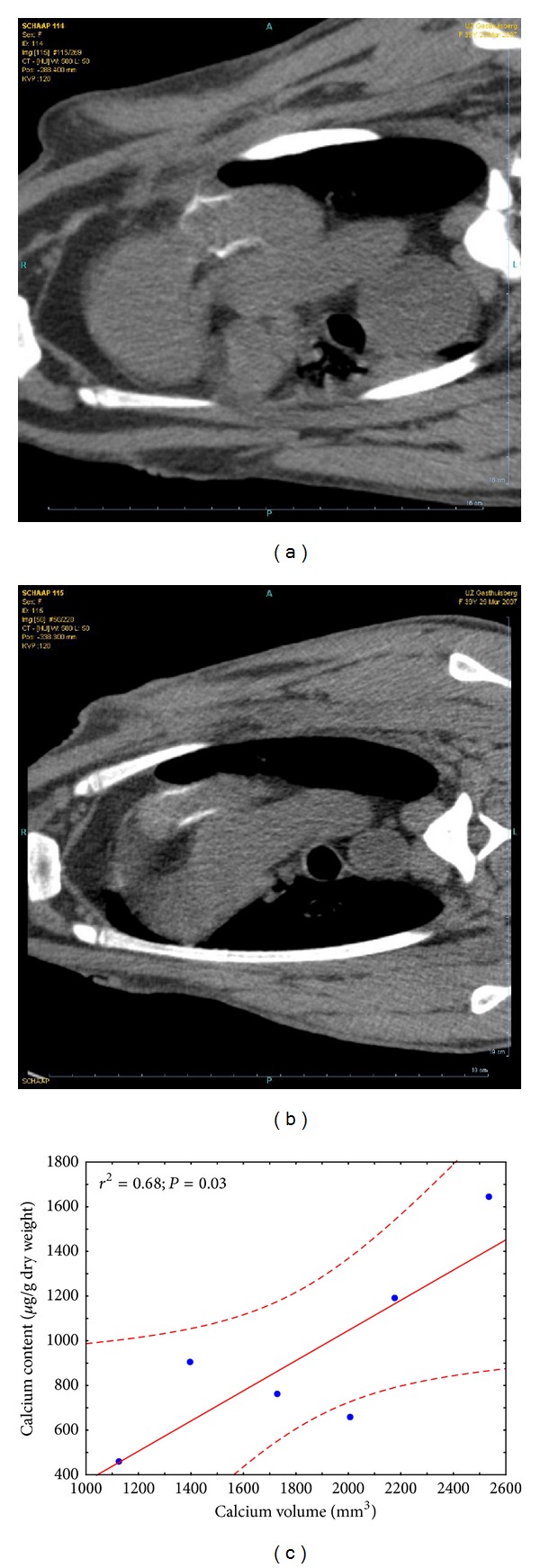
(a) and (b) show two representative examples of CT scans performed at 20 weeks. Clear mineralization of the wall portion is visible. Through further image processing, the region of the implanted valve can be selected. Determination of calcium volume is then performed, with calcium defined as every sample >100 and <400 HU. (c) shows the correlation between this CT-based calcium volume determination (in mm³) and the calcium content (*μ*g/g dry weight) as measured with the spectrophotometer.

At the 12-week scans, the animals already revealed important mineralization in the wall portion of the implanted stentless conduit. Based on the CT images at 12 weeks, a calculated mean calcium volume of 1373 ± 388 mm³ was obtained. This wall mineralization increased visually on the 20-week scans. The mean calculated calcium volume increased to 1831 ± 581 mm³ (*P* = 0.09 versus scans at 12 weeks). Figures 1(a) and 1(b) show two representative examples of obtained scan images at 20 weeks. Through further image processing, the calcified bioprosthesis can be virtually isolated from the surrounding tissues, resulting in images that enable examination of the distribution of calcium in 3D and quantification of the mineralization based on the measured Hounsfield units. Faxitron and histology confirmed important tissue mineralization of the porcine aortic wall portion. The measured calcium content values of the aortic wall portion (by spectrophotometry) were clearly pathologically elevated: mean calcium content was 89.8 *μ*g/mg, ranging from 47.1 to 146.3. After correction for the weight of the whole valve, a positive correlation could be demonstrated with the calcium content values as determined by the CT scan at 20 weeks (Figure 1(c)).

### 3.2. Mitral Model (Stented Pericardial Valves in Mitral Position)

**Figure 2:**
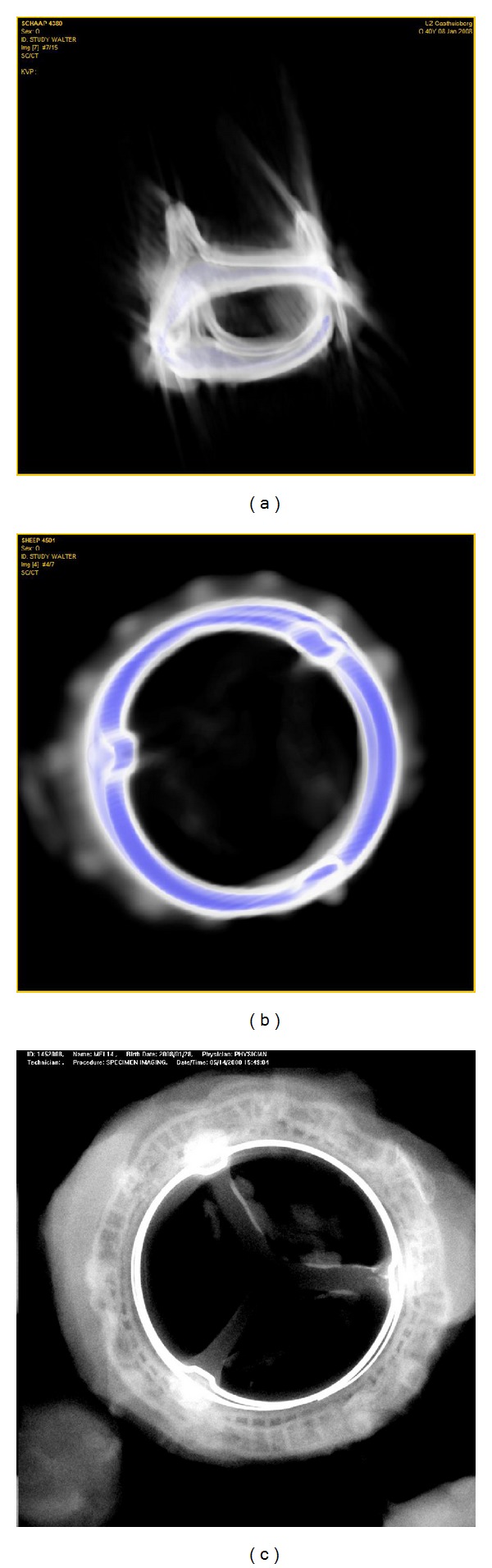
(a) shows a reconstructed image from a scan performed in the mitral model. The alloy frame of the valve stent produces, probably due to its continuous motion, important scattering around the prosthesis. (b) illustrates how this scattering can be filtered, but through this process potential areas of mineralization within the cusps are also deleted. (c) shows the Faxitron X-ray image after explantation at 20 weeks, illustrating that there is clear calcification within two of the commissural areas.

The images obtained from the stented valves in mitral position showed important scattering, most likely as a result from the continuously moving alloy frame (Figure 2(a)). Attempts were made to diminish the presence of this scattering by repeating the scan on a Philips Brilliant-64 scanner with ECG triggering, which did not result in a better scan quality. Despite the difficult image interpretation, all scans were performed to allow comparison of all images. We were unable to detect any signs of cusp mineralization based on the processed CT images, although the postexplant Faxitron X-ray analysis clearly demonstrated cusp mineralization after 20 weeks (Figure 2(b)). Mean measured calcium content in the cusps was 13.81 ± 17.49 *μ*g/mg dry weight, ranging from 1.71 to 65.39 *μ*g/mg. The highest values were obtained at the commissures and the base of the cusps. After explantation, a new scan from the explanted valves was repeated, which did not result in scattering and artifacts and where the calcific residues could be observed.

### 3.3. Patch Model (Pericardial Patches in the Wall of the Neck Vessels)

**Figure 3:**
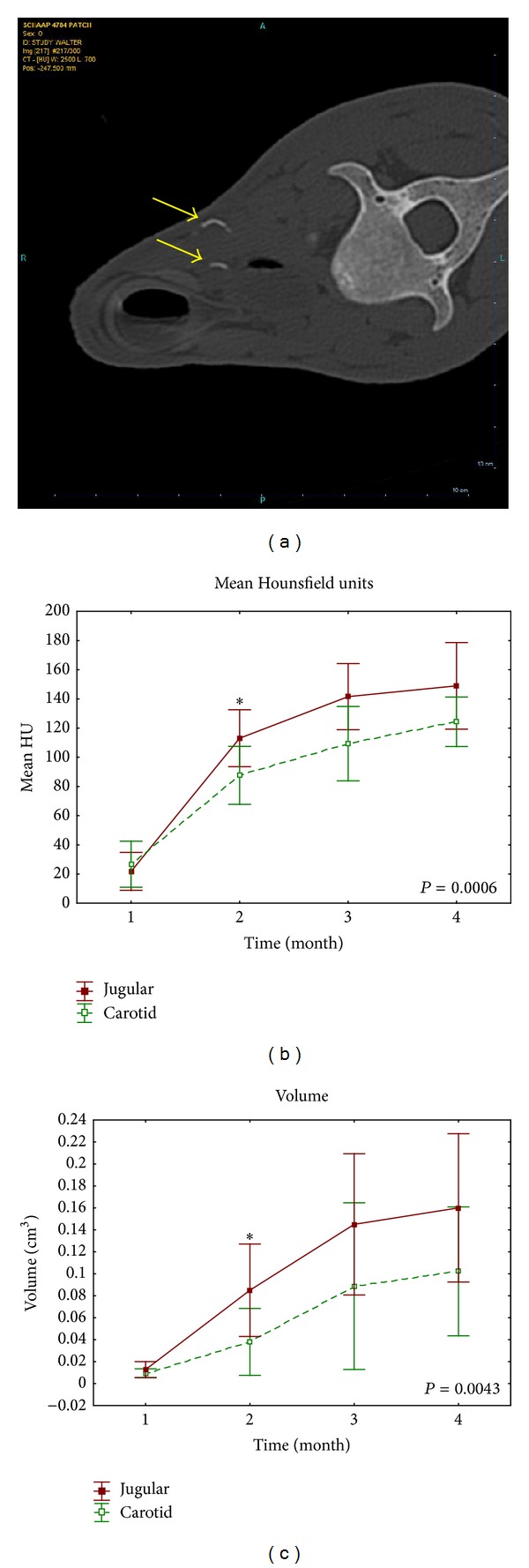
(a) shows a representative example of the CT scan obtained in the patch model at 4 months. Calcification of the patches in both venous position (upper arrow) and arterial position (lower arrow) is visible. (b) and (c) show the evolution of mean HU and mean calcium volume over time, in both the venous and arterial samples.

At the 4-week scan, no mineralization was visible in any of the implanted samples. In the second scan, at 8 weeks, clear beginning calcification was detectable in several patches.  Figure 3 shows the evolution of mean HU (Figure 3(a)) and calcified volume (Figure 3(b)) as determined by the scan images throughout the different scans. Tissue calcification starts at 8 weeks after implantation and evolves further in time. In the venous samples, mean HU increased from 21.8 ± 13.0 HU at 4 weeks to 113.1 ± 19.5 HU at 8 weeks (*P* < 0.001). The arterial samples showed a similar evolution: 26.7 ± 15.8 HU at 4 weeks rising to 87.6 ± 19.9 HU at 8 weeks (*P* < 0.001). At 20 weeks, the mean HU were 148.9 ± 29.6 and 124.4 ± 16.9 in the venous and arterial samples, respectively. The mean calculated calcium contents went up to 160.0 ± 42.4 mm³ in the venous samples and 102.2 ± 36.8 mm³ in the arterial samples (*P* = 0.08, taking into account that the venous samples were also larger in actual size). Based on the CT results, we were unable to detect a significant difference in level of calcification between the venous and arterial implants, both in amount of calcification as in time evolution. After explantation, Faxitron X-rays showed clear mineralization in all samples. Measured calcium content values were slightly higher for the carotid implants (25.1 ± 18.6 *μ*g/mg) versus the venous implants (13.1 ± 5.8 *μ*g/mg), but this difference was not significant (*P* = 0.07).

## 4. Discussion

The main limitation of bioprosthetic heart valves is still the limited tissue durability. Calcification of the glutaraldehyde-fixed tissue is, amongst other factors, one of the main reasons for biological valve failure over time, certainly when implanted in younger recipients. Both the valve industry and several research centers worldwide invest time and effort trying to improve the durability of biological valvular tissue, either by changes in valve design or in tissue treatments. Such new experimental valves or new treatments need to be evaluated in reliable animal models before a clinical application can be conceived. Current animal models of bioprosthetic tissue mineralization offer information at the moment of explantation of the tested sample. There is no information on the calcification status of the tissue during implantation. An animal model offering reliable and reproducible tissue mineralization with the possibility of noninvasive, *in vivo* evaluation (and quantification) of tissue mineralization would be a step forward. 

In this study, we evaluated the ability of high-resolution CT scanning to detect *in vivo* tissue mineralization in three known models of valvular tissue calcification. The RVOT model for implantation of stentless grafts or even homografts is known to produce important mineralization in the wall of the implanted conduits [2, 7]. This wall mineralization is a phenomenon observed clinically as well [9, 10]. Wall mineralization limits the use of stentless porcine valves in RVOT reconstructive surgery, and it is also known to be one of the factors involved in the degeneration of aortic and pulmonary homografts in that position. Our experience with the RVOT model clearly shows the degenerative modes in both stentless xenografts and homografts [2]. The multislice CT scanning enables visualization and quantification of the distribution and extent of tissue mineralization in this model. A new valve design or new treatment aiming at reducing wall mineralization in a graft for RVOT reconstruction could be tested using this methodology. Cusp calcification, which is known to be still limited in these grafts at 5 months, was not visualized, but it would be feasible to expand the implant duration beyond 6 months or 1 year, to further study graft behavior and degeneration.

In the mitral model, we were unable to detect tissue calcification of the cusps in stented bioprosthesis. This is due to the (continuously) moving metal frame of the valve stent. Despite the fact that clear commissural calcific aggregates were present, this could not be visualized nor quantified in the serial CT scan images. Further image processing can diminish the presence of these artifacts, but this manipulation most likely also impedes detection of any soft tissue mineralization in the cusps or at the commissures. The alloy used for stent construction does not produce artifacts when scanned *ex vivo* (in still position), but *in vivo*, in a beating heart, the alloy produces important scattering. Efforts to reduce the animals' heart rate and change to ECG-triggered scans did not influence this phenomenon.

Finally, in the more simplified patch model, the mineralization of several tissue patches could be detected nicely. Not only the end stage of implantation is studied, but the kinetic process of the tissue mineralization can be captured. This model enables the study of tissue mineralization over time using repeated CT scans, and it enables the direct comparison of differentially treated tissue samples implanted in one animal. This eliminates the important inter individual variation that is known to occur in animal experiments with biological tissue. This model would be useful to evaluate new tissue treatments in comparison to currently used fixation methods and postfixation antimineralization treatments. Obviously, the model is not reliable for valve evaluation in total since the tissue does not serve as a valve. Differences between venous and arterial implants were small as previously described. The arterial implants have a tendency for slightly higher calcium contents, but the venous implants have the advantage that larger patches can be used in the implantation.

It is possible to detect evolving bioprosthetic tissue calcification in a noninvasive way, using different models of valvular tissue implantation. Current juvenile sheep models of stentless valve implantation in RVOT position offer the possibility of intermediate tissue evaluation using serial, multislice CT scanning. Similarly, ongoing tissue calcification can be visualized and quantified in a simplified model of patch implantation in the neck vessels of young sheep. The simplified model has the possibility of a direct comparison between differentially treated samples within one animal. Not only the end stage of implantation is studied, but the kinetic process of the tissue mineralization can be captured. The alloy in stented pericardial valves however disturbs the image quality considerably through scattering.
